# Creating web applications for spatial epidemiological analysis and mapping in R using Rwui

**DOI:** 10.1186/1751-0473-6-6

**Published:** 2011-04-01

**Authors:** Richard Newton, Andrew Deonarine, Lorenz Wernisch

**Affiliations:** 1 MRC Biostatistics Unit, Robinson Way, Cambridge, CB2 0SR, UK; 2 MRC Laboratory of Molecular Biology, Hills Road, Cambridge, CB2 0QH, UK

## Abstract

**Background:**

Creating a user friendly web based application which executes an R script allows physicians, epidemiologists, and others unfamiliar with the statistical language to perform powerful statistical analyses easily. The geographic mapping of data is an important tool in spatial epidemiological analysis, and the R project includes many tools for such analyses, but few for visualization. Hence, web applications that run R for epidemiological analysis need to be able to present the results in a geographic format.

**Results:**

Rwui is a web application for creating web based applications for running R scripts. We describe updates to Rwui that enable it to create web applications for R scripts which return the results of the analysis to the web page as geographic maps.

**Conclusions:**

Rwui enables statisticians to create web applications for R scripts without the need to learn web programming. Creating a web application provides users access to an R based analysis without the need to learn R. Recent updates to Rwui have increased its applicability in the field of spatial epidemiological analysis.

## Background

Spatial epidemiology combines the geographic mapping of disease distributions with pattern analysis, spatial statistics and disease modelling [1-3]. The statistical language R [4] is a high-level mathematical scripting language which includes a vast range of pre-programmed functions, and facilitates rapid development of data analysis programs. It contains many functions useful for epidemiological analysis, including three dedicated epidemiological packages Epi [5] and Epitools [6] and SpatialEpi [7].

Statisticians creating epidemiological analyses in R may need to make an analysis available to users who are unfamiliar with the language. A solution is to provide a user-friendly web application for the R script. Values for variables and data files for processing are submitted by the user on a web form. The web application then runs the R script on a server, out of sight of the user, and returns the results of the analysis to the user's web page.

Rwui (R Web User Interface) [8] is a web application that creates web applications for running R scripts. Code for the web application is generated automatically so that a fully functional web application for an R script can be implemented in a matter of minutes. So statisticians who are unfamiliar with web programming can easily create web applications for running R scripts.

There have been several updates to Rwui since it was first published [8], permitting the applications created by Rwui to include a number of new features, the most important being:

• Applications can be 'daisy-chained', the output of one web application forming the input to another web application.

• The results of an analysis can be e-mailed to the user rather than displayed on the web page.

• Image maps can be added to graphical output. Adding an image map makes the image 'clickable', so that supplementary information can be displayed according to the spatial coordinates where the image is clicked.

• The results of the R analysis can be displayed on a geographic map.

This article describes the last of these new features, namely displaying results on geographic maps. The geographic mapping of data has long been a vital tool in epidemiological analysis. So web applications that run R for epidemiological analyses should be able to present the results in a geographic format. Integrating the Google maps project [9] into Rwui has made this possible in the web applications that Rwui creates.

## Implementation

### Creating web applications with Rwui

**Figure 1:**
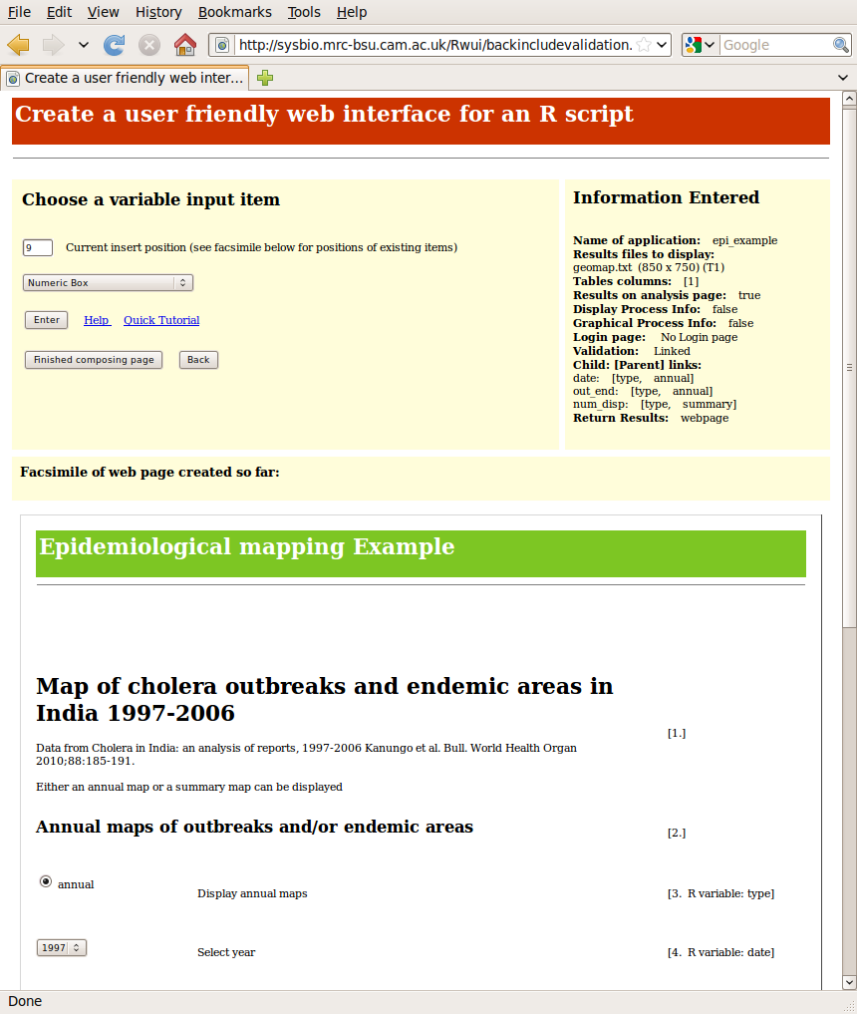
**Screenshot of one web page of Rwui**. Screenshot of one web page of Rwui, on which input items are added. A facsimile of the epidemiological example application being created can be seen on the lower half of the page.

The information that Rwui needs in order to create a web application for running an R script is entered on a series of web forms. Using these forms the statistician designs the web application they wish to construct. After entering a title and introductory text for the application, the input items that will appear on the application's web page are selected. Input items may be, for example, Numeric or Text entry boxes, Checkboxes, Drop-down lists, Radio Buttons and File Upload boxes. Figure 1 shows a screenshot of this page of Rwui during the design of an epidemiological example application. Each of the input variables of the R script (i.e those variables that require a value supplied by the user) must have a corresponding input item on the application's web page. Various other options relating to the function and layout of the application being created can then be selected on a further sixteen web forms. Rwui displays a facsimile of the web page that has been created as items are added to the page. After the R script has been uploaded, Rwui generates the web application for the script, which can be downloaded as a zip or tgz file.

### Installing web applications created by Rwui

The completed applications will run on a Tomcat server [10]. Tomcat is free and widely used server software, easy to install on both Unix and Windows machines. All that needs to be done to use the downloaded web application is to place the completed application's .war file in the Tomcat server's webapps directory. As well as enabling remote access to applications on a server, Tomcat can be installed on stand-alone machines, in which case the web applications are accessed in a browser on the machine via the 'localhost' URL.

### Using web applications created by Rwui

Users of web applications created by Rwui simply enter the information they wish to analyse on the web page via the input items on the page, such as Numeric entry boxes or File Upload boxes, and then press the Analyse button. The information they have entered is sent to the server where the application has been installed and the R script is run on the uploaded information automatically. Once finished, the results are returned to the user's web page. As well as displaying the results on the data entry web page a unique results page is created for each data submission from which the results of the analysis can be downloaded. If the script takes some time to run, the web application can be designed to display progress information for the user and may also include a cancel button if required.

### The structure of web applications created by Rwui

The web applications created by Rwui are Java based web applications that use the open source Apache Struts framework [11]. The R script is run using R batch mode. The batch command is placed in a shell script, which is run as a Process using the application's instance of the Runtime class. A Java application has one instance of the Runtime class, which allows the application to interface with the environment in which it is running. The Process class has a method that causes the current thread to wait until the Process has completed. Before the R script is run, the values of the variables that the user entered on the web pages are passed to the R script. The application writes this information, as R assignments, into a text file which is concatenated with the main R script prior to execution.

The application waits for the script to finish and then displays the results on the web page. A uniquely named working directory is created each time the R script is run. To pass results back to the web page, the R script writes results to this directory. The web application, on completion of the R script, displays the files in this directory on the Results page.

### Displaying geographic maps in web applications created by Rwui

A web application created by Rwui can display the results of the R analysis as a geographic map, by displaying a static Google map (or maps). A typical application would have the user of the web application uploading a data file. The data is then analysed by the R script on the server. The R script produces a text file of results (eg. called geomap.txt). These results are then displayed for the user on the web page as a geographic map. The format of the text file of results produced by the R script is important. Each line of the results file must contain the name of one valid static Google maps parameter followed by the value for that parameter. Valid parameter names are documented in the Google static maps guide [9]. The parameter name and the parameter value must be separated by an equals (=) sign. An example results file would be:

center=India

zoom=5

size=640x640

maptype=roadmap

style=feature:road|visibility:off style=feature:poi.park|visibility:off

style=feature:administrative.province|element:geometry|hue:0x0000000|saturation:-100|lightness:-100

path=color:0xffff00CC|weight:20|16.26,79.15|16.36,79.15

path=color:0xff0000AA|weight:80|26.31,92.11|26.41,92.11

path=color:0xff8000AA|weight:40|21.59,82.51|21.69,82.51

path=color:0xffff00CC|weight:20|22.93,71.50|23.03,71.50

path=color:0xffff00CC|weight:20|28.97,76.47|29.07,76.47

path=color:0xffff00CC|weight:20|31.94,77.26|32.04,77.26

path=color:0xffff00CC|weight:20|14.56,75.81|14.66,75.81

path=color:0xffff00CC|weight:20|10.14,76.55|10.24,76.55

path=color:0xffff00CC|weight:20|23.18,78.14|23.28,78.14

path=color:0xffff00CC|weight:20|19.14,75.81|19.24,75.81

path=color:0xdf7070AA|weight:60|20.67,84.93|20.77,84.93

path=color:0xffff00CC|weight:20|30.67,75.63|30.77,75.63

path=color:0xffff00CC|weight:20|10.98,78.53|11.08,78.53

path=color:0xdf7070AA|weight:60|23.38,91.63|23.48,91.63

path=color:0xdf7070AA|weight:60|22.85,87.72|22.95,87.72

sensor=false

**Figure 2:**
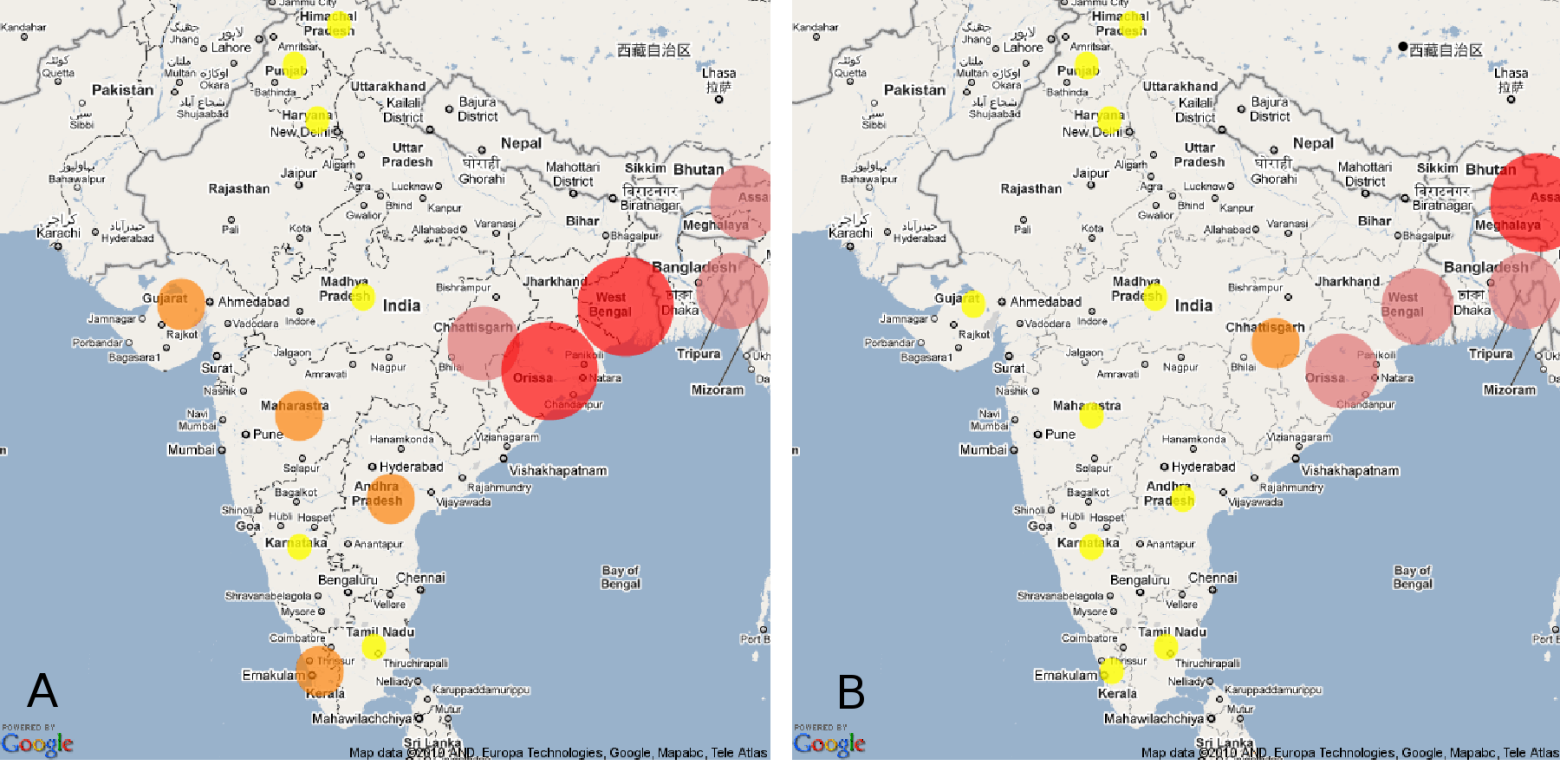
**Screenshot of maps from example application**. Maps from the example application created by Rwui. Map A shows the number of people affected by cholera outbreaks in different states of India between 1997 and 2006 (red > 50000, pink > 5000, orange 500, yellow < 500). Map B shows the percentage of each state's population affected by the outbreaks (red > 0.5%, pink > 0.05%, orange > 0.005%, yellow < 0.005%).

The above text file of results generated the geographic map seen in Figure 2B.

To include a geographic map when designing a web application with Rwui, it is simply a matter of ticking a checkbox on the appropriate web form asking whether the file to display on the results page is a geographic map, and entering the name of the text file of results that the R script will generate (eg. geomap.txt). The R script which the web application is designed to run must of course include code for generating a file called geomap.txt containing the appropriate parameter name/value pairs to produce the desired geographic map.

## Results

An example web application created by Rwui which displays epidemiological information on a geographic map is available at http://sysbio.mrc-bsu.cam.ac.uk/epi_example. The R script is available at http://sysbio.mrc-bsu.cam.ac.uk/epi_example/epi_example.R. Figure 2 shows screenshots of geographic maps from this example application. The application displays data on cholera outbreaks in India between the years 1997 and 2006. This data was taken from a paper by Kanungo et al. [12]. In this simple demonstration application the data to be analysed is held on the server, but in more general use an epidemiological application created by Rwui could upload user data by means of a File upload or Zip-file upload box on the web page. The R script in this example parses the data and performs a very simple data analysis, but in general, besides parsing the data, any of the many statistical analysis methods available in R could be applied to the data prior to display as a geographic map.

## Conclusions

Rwui enables statisticians to create web applications for their R scripts without the need to learn web programming. Web applications for R scripts provide users access to R based analyses without the need to learn R. Including Google static maps into the display options for web applications created by Rwui has increased its applicability in the field of spatial epidemiological analysis. Further developments to Rwui will concentrate on four areas. Firstly, making the geographic maps interactive, so that regions can be defined on a map by mouse clicks from the user, and statistics then calculated for the defined areas by R and returned to the user on the web page. Secondly, integrating the Google dynamic Javascript maps API [13] which allows the user to zoom and pan and includes many additional capabilities for marking and annotating maps. Thirdly, producing R functions to aid creating the file of parameter name/value pairs required for a particular design of map. Fourthly, producing a downloadable version of the complete Rwui project for local installation.

## Availability and requirements

• Project name: Rwui

• Project home page: http://sysbio.mrc-bsu.cam.ac.uk/Rwui

• Programming language: Java, JSP, Struts, Javascript

• License: None

• Any restrictions to use by non-academics: None

The web applications created by Rwui require:

• Operating system(s): Linux or Windows

• Other requirements:

- Java 1.5.0 or higher

- Tomcat 5.5 or higher

- An R version compatible with the R script

• License: GNU GPL for R, and if the Google maps facility is used, its license [14].

• Any restrictions to use by non-academics: If the Google maps facility is used, its terms [14].

## Competing interests

The authors declare that they have no competing interests.

## Authors' contributions

RN and LW designed and programmed Rwui. AD contributed the concept of the geographic mapping facility and to its design. All authors read and approved the final manuscript.
